# Supplementary material for: Obstructive Sleep Apnea and Acute Lower Respiratory Tract Infections: A Narrative Literature Review
Source: Antibiotics (Basel). 2024 Jun 6;13(6):532. doi: 10.3390/antibiotics13060532 (PMC11200551; doi:10.3390/antibiotics13060532)
Supplement: Supplementary file 1 [file antibiotics-13-00532-s001.zip › antibiotics-3012007-supplementary.pdf]

Supplementary material: Obstructive Sleep Apnea and Acute Lower Respiratory Tract Infections: A Narrative Literature Review

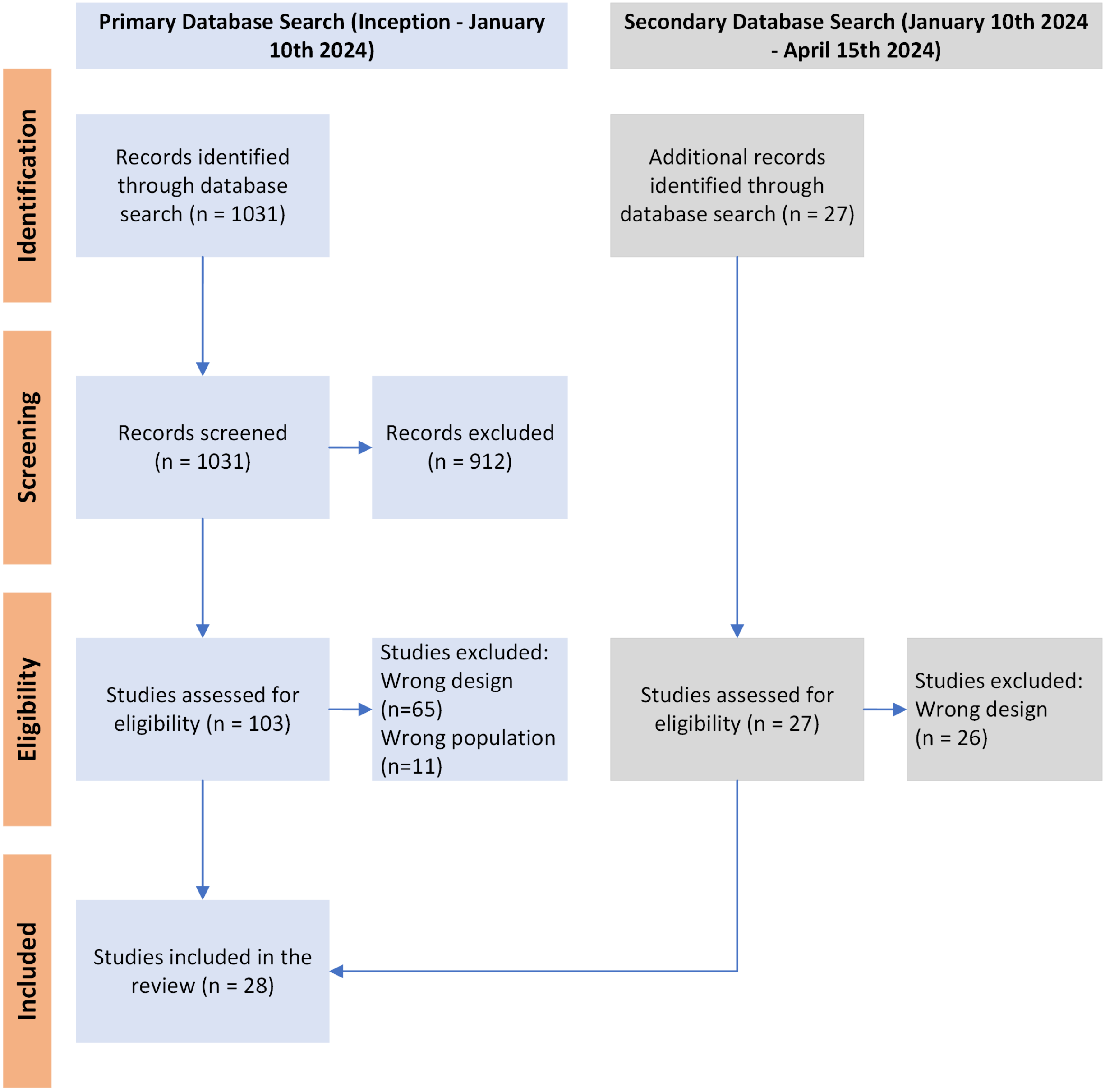

Figure S1. Flowchart of Study Selection.

Abbreviations: n: number of studies.
